# Preventing Antibiotic-Resistant Infections: Additively Manufactured Porous Ti6Al4V Biofunctionalized with Ag and Fe Nanoparticles

**DOI:** 10.3390/ijms232113239

**Published:** 2022-10-31

**Authors:** Niko E. Putra, Marius A. Leeflang, Verena Ducret, Viorica Patrulea, Lidy E. Fratila-Apachitei, Karl Perron, Hua Ye, Jie Zhou, Iulian Apachitei, Amir A. Zadpoor

**Affiliations:** 1 Department of Biomechanical Engineering, Faculty of Mechanical, Maritime, and Materials Engineering, Delft University of Technology, Mekelweg 2, 2628 CD Delft, The Netherlands; 2 Microbiology Unit, Department of Botany and Plant Biology, University of Geneva, 30 Quai Ernest-Ansermet, 1211 Geneva, Switzerland; 3 Institute of Biomedical Engineering, Department of Engineering Science, University of Oxford, Oxford OX3 7DQ, UK; 4 Institute of Pharmaceutical Sciences of Western Switzerland, University of Geneva, 1 Rue Michel Servet, 1211 Geneva, Switzerland; 5 Section of Pharmaceutical Sciences, University of Geneva, 1 Rue Michel Servet, 1211 Geneva, Switzerland

**Keywords:** antibiotic-resistant bacteria, implant-associated infection, surface biofunctionalization, additive manufacturing, antibacterial, iron nanoparticles, silver nanoparticles

## Abstract

Implant-associated infections are highly challenging to treat, particularly with the emergence of multidrug-resistant microbials. Effective preventive action is desired to be at the implant site. Surface biofunctionalization of implants through Ag-doping has demonstrated potent antibacterial results. However, it may adversely affect bone regeneration at high doses. Benefiting from the potential synergistic effects, combining Ag with other antibacterial agents can substantially decrease the required Ag concentration. To date, no study has been performed on immobilizing both Ag and Fe nanoparticles (NPs) on the surface of additively manufactured porous titanium. We additively manufactured porous titanium and biofunctionalized its surface with plasma electrolytic oxidation using a Ca/P-based electrolyte containing Fe NPs, Ag NPs, and the combinations. The specimen’s surface morphology featured porous TiO_2_ bearing Ag and Fe NPs. During immersion, Ag and Fe ions were released for up to 28 days. Antibacterial assays against methicillin-resistant *Staphylococcus aureus* and *Pseudomonas aeruginosa* showed that the specimens containing Ag NPs and Ag/Fe NPs exhibit bactericidal activity. The Ag and Fe NPs worked synergistically, even when Ag was reduced by up to three times. The biofunctionalized scaffold reduced Ag and Fe NPs, improving preosteoblasts proliferation and Ca-sensing receptor activation. In conclusion, surface biofunctionalization of porous titanium with Ag and Fe NPs is a promising strategy to prevent implant-associated infections and allow bone regeneration and, therefore, should be developed for clinical application.

## 1. Introduction

The emergence of antibiotic-resistant bacteria, such as methicillin-resistant *Staphylococcus aureus* (MRSA), has given rise to an increasing number of untreatable orthopedic implant-associated infections and an increase in patient mortality [1,2]. In addition to the *Staphylococcus* genus, *Pseudomonas aeruginosa (P. aeruginosa)* is one of the main pathogenic Gram-negative species isolated from bone implants [3]. Once bacteria adhere to the implant surface, biofilm formation follows [4,5]. Biofilm significantly lowers the efficacy of antibiotics, even if they are administered locally [6,7]. As more antibiotic-resistant bacteria appear every year, the choice of antibiotics for treatment becomes increasingly limited [8,9]. This challenges current therapeutic approaches and necessitates the development of novel strategies to combat such infections.

To minimize the risk of implant-associated infection, it is essential to eradicate the bacteria within days after the conclusion of the surgical procedure [10]. This will minimize the number of potential adherent bacteria and their chance to form biofilms. In addition, speeding up the osseointegration of the implants can assist in covering the surfaces of implants by the host tissue’s extracellular matrix, leaving less surface area for bacteria to occupy. Orthopedic implants possessing such dual functionalities (i.e., being both bactericidal and osteoconductive), have been made possible by additive manufacturing (AM), followed by a surface biofunctionalization step [11]. Highly porous implants with bone-mimicking mechanical properties can also promote new bone formation [12,13]. Such implants usually have a very large surface area, which can be biofunctionalized using combinations of powerful antibacterial agents against pathogenic bacteria and bioactive agents to aid bone regeneration [14,15,16].

Choosing the right antibacterial agent is paramount. Ag is shown to be effective against multidrug-resistant bacteria [17,18]. However, it is crucial to properly tune the Ag dose, as its therapeutic window between bactericidal and cytotoxic activity is very narrow [19]. Recent studies have reported discouraging in vivo results of bone implants with Ag-doped coatings [20,21], despite positive in vitro outcomes. High Ag concentration may weaken the initial immune response in vivo by impairing the activity of neutrophils [22]. Likewise, Ag has been found to cause abnormal in vivo bone remodeling in non-sterile environments [22]. To overcome such challenges, the combination of Ag with other elements or compounds has been explored to achieve the best bactericidal activity while simultaneously improving the response of the host tissue [11,16,23].

To date, no study has been performed on immobilizing both Ag and Fe nanoparticles (NPs) on the surface of AM porous titanium, despite that Fe-based NPs have been widely used in other biomedical applications, such as magnetic field-guided antimicrobial therapy [24,25]. Fe-based NPs are often used in combination with other compounds [26,27,28] to achieve an improved antimicrobial efficacy synergistically. Fe ions released from NPs are able to catalyze the generation of highly reactive hydroxyl radicals that increase oxidative stress, ultimately promoting bactericidal activity [29,30,31]. In addition, the high affinity of Fe towards P may attract more phosphate ions onto the implant surface, favoring osteoblast adhesion [32]. We hypothesized that by biofunctionalizing the porous titanium surface using a lower Ag NPs concentration in the presence of Fe NPs, the implants would preserve the bactericidal properties due to the synergistic effects of these NPs. Meanwhile, a reduced Ag NPs concentration could decrease the potential cytotoxic effects of Ag [33].

We prepared porous titanium scaffolds using selective laser melting (SLM), followed by a plasma electrolytic oxidation (PEO) process involving a Ca/P-containing electrolyte doped with Ag and Fe NPs. During the PEO process, Ag and Fe NPs were immobilized on the porous TiO_2_ surface of the scaffolds [34,35]. The biofunctionalized porous oxide layer contained embedded Ca/P ions that are shown to promote osteogenesis [36,37]. Following PEO treatment, we evaluated the in vitro antibacterial activity of the implants against MRSA and *P. aeruginosa*, and assessed their in vitro cytocompatibility and phospho-calcium-sensing receptor (phospho-CaSR) Thr888 activity in preosteoblast MC3T3-E1 cells.

## 2. Results

### 2.1. Surface Biofunctionalization of Ti6Al4V Scaffolds

**Figure 1:**
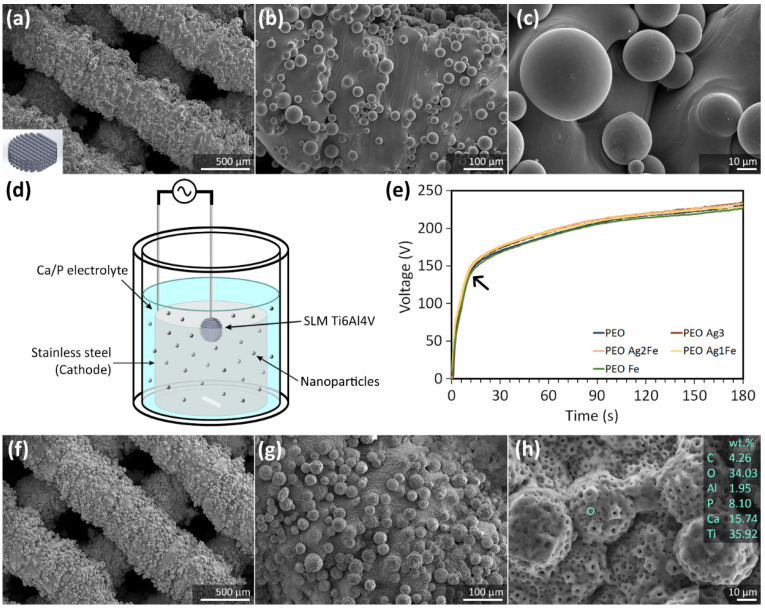
(**a**–**c**) The surface morphology of selective-laser-melted Ti6Al4V scaffolds at different magnifications, imaged using SEM. (**d**) An illustration of the PEO setup with the titanium scaffold and the stainless steel cathode in the Ca/P-containing electrolyte doped with NPs for surface biofunctionalization. (**e**) The *V-t* transients were recorded during the surface biofunctionalization of the titanium scaffolds using the electrolytes containing Fe NPs and Ag NPs of varying concentrations. (**f**–**h**) The surface morphology of the PEO scaffold groups after being subjected to 180 s of surface biofunctionalization were imaged at different magnifications using SEM and analyzed with EDS.

The titanium scaffolds exhibited partially molten powder particles firmly attached to the surface of the struts (Figure 1a–c) with an absolute porosity of 48.8 ± 0.6%. The surface of the scaffolds was biofunctionalized using the PEO setup in the Ca/P electrolyte solutions doped with Ag and Fe NPs (Figure 1d). During the PEO process, the *V-t* curves of all the scaffold groups were alike (Figure 1e). At the start, the voltage increased at a rate of 13 ± 1 V/s up to the dielectric breakdown point at 130 ± 4 V, where the plasma discharge started. Afterward, the voltage continued to rise at a slower rate of 0.57 ± 0.04 V/s (Figure 1e). After the PEO process, a uniformly distributed micro-/nano-porous TiO_2_ layer on the titanium surface was observed (Figure 1f–h). The absolute porosity of the biofunctionalized scaffolds did not change significantly: 47.9 ± 1.1% for PEO, 48.0 ± 0.5% for PEO Ag3, 47.7 ± 0.3% for PEO Ag2Fe, 48.0 ± 0.4% for PEO Ag1Fe, and 47.6 ± 0.8% for PEO Fe. The porous biofunctionalized layer without NPs contained C, O, Ca, P, Ti, and Al (Figure 1h).

**Figure 2:**
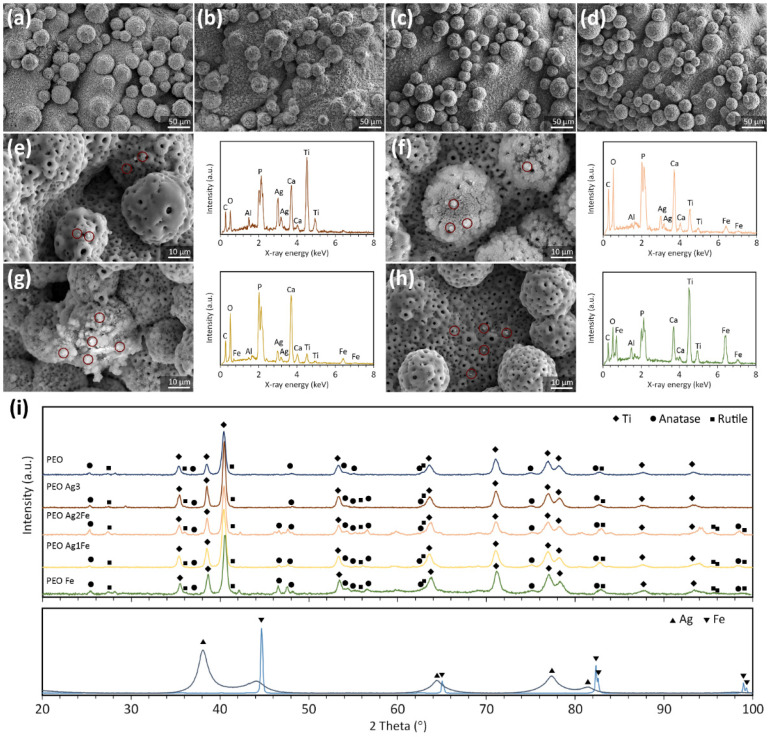
The surface morphology of (**a**) PEO Ag3, (**b**) PEO Ag2Fe, (**c**) PEO Ag1Fe, and (**d**) PEO Fe scaffold groups after 180 s of surface biofunctionalization, imaged using SEM. The chemical compositions of the biofunctionalized scaffolds containing Ag and/or Fe NPs: (**e**) PEO Ag3, (**f**) PEO Ag2Fe, (**g**) PEO Ag1Fe, and (**h**) PEO Fe. The circles indicate the locations of EDS analysis. (**i**) The phase compositions of the biofunctionalized scaffolds and Ag and Fe NPs.

SEM confirmed that the additions of Ag and Fe NPs to the electrolyte did not change the surface morphology of the biofunctionalized scaffolds (Figure 2a–d). NPs were tightly embedded in the porous TiO_2_ surface layer. Moreover, Ag NPs can be identified on PEO Ag3 specimens (Figure 2e), Ag and Fe NPs on PEO Ag2Fe (Figure 2f), PEO Ag1Fe specimens (Figure 2g), and Fe NPs on PEO Fe specimens (Figure 2h). 

In addition to Ag and Fe, a number of other elements, including C, O, Ca, P, Ti, and Al, were detected on the surface of the scaffolds. Interestingly, the Ag and Fe NPs morphology on PEO Ag2Fe and PEO Ag1Fe groups appeared to be in the form of clusters (Figure 2f–g). An EDS spot analysis on the Ag and Fe NPs clusters revealed the surface chemistry being rich in Ca and P (Figure 2f–g), compared to that on Ag NPs and Fe NPs alone (Figure 2e,h). Furthermore, the XRD analysis of the biofunctionalized scaffolds revealed the presence of a mixture of rutile and anatase phases in the crystalline TiO_2_ surface layer on all the scaffold groups (Figure 2i). The original phases of Ag and Fe NPs were not observed. The anatase peaks at 2 Theta of 46–48° and 54–57° and the rutile peaks at 2 Theta of 96–100° were more pronounced on the scaffold groups containing Fe NPs (Figure 2i).

### 2.2. Ag, Fe, and Ca Ion Release Kinetics

**Figure 3:**
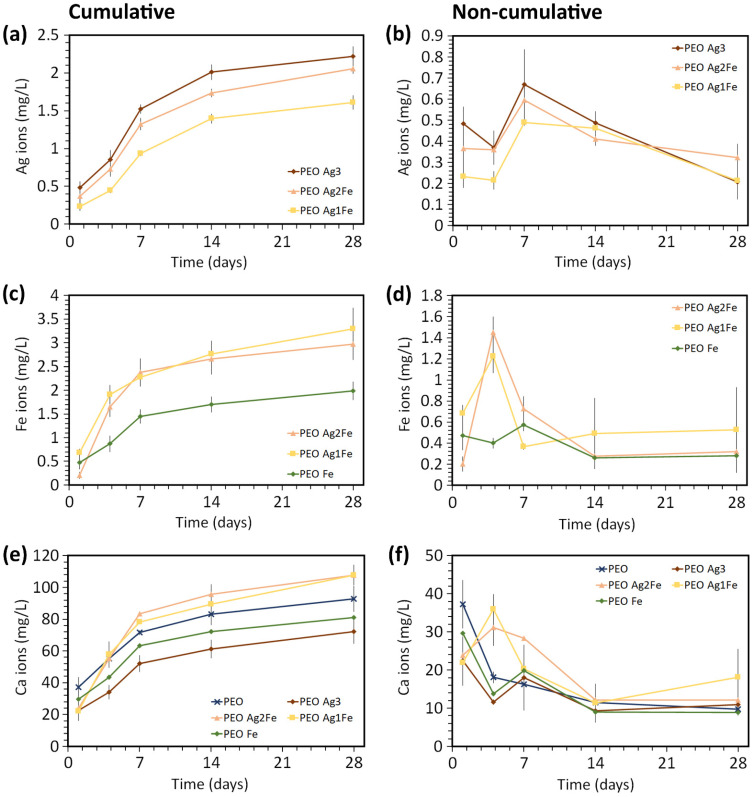
The (**a**,**c**,**e**) cumulative and (**b**,**d**,**f**) non-cumulative (**a**,**b**) Ag, (**c**,**d**) Fe, and (**e**,**f**) Ca ion concentrations released from the surface-biofunctionalized scaffolds during immersion over 28 days.

The surface-biofunctionalized scaffolds released Ag, Fe, and Ca ions during the immersion test for up to twenty-eight days, with the highest release rates during the first 7 days (Figure 3). After 1 day of immersion, PEO Ag3 specimens released 1.3 and 2.1 times more Ag ions compared to PEO Ag2Fe (n.s., *p* > 0.05) and PEO Ag1Fe (*p* < 0.01) specimens (Figure 3a). The cumulative Ag ion release from PEO Ag3 specimens was the highest throughout the immersion period, followed by that from PEO Ag2Fe and PEO Ag1Fe groups. In addition, the highest concentration of Ag ions was released on day 7 for all scaffold groups (Figure 3b).

The combination of Ag and Fe NPs enhanced the release of Fe ions (Figure 3c,d). In comparison with PEO Fe specimens, the cumulative Fe ions released from PEO Ag2Fe were 1.9 times higher (*p* < 0.001) and 2.2 times higher (*p* < 0.0001) than PEO Ag1Fe on day 4 (Figure 3c). After 28 days of immersion, the cumulative concentrations of Fe ions from the PEO Ag2Fe and PEO Ag1Fe groups were still 1.5 and 1.7 times higher, respectively, than that from the PEO Fe group (Figure 3c). The highest concentration of Fe ions was released on day 4 for PEO Ag2Fe and PEO Ag1Fe groups (Figure 3d). Meanwhile, the PEO Fe group exhibited a stable concentration of Fe ion release over time (Figure 3d).

Furthermore, PEO specimens released the largest amount of Ca ions in the first 24 h (*p* > 0.05, Figure 3e). At other time points, the cumulative Ca ions released from PEO Ag2Fe and PEO Ag1Fe groups were comparable to those from PEO specimens (*p* > 0.05), but higher than those from PEO Ag3 (*p* < 0.0001) and PEO Fe specimens (*p* < 0.001, Figure 3e). The highest concentration of Ca ions was released on day 1 for the PEO, PEO Ag3, PEO Fe groups, and on day 4 for the PEO Ag2Fe and PEO Ag1Fe groups (Figure 3f).

### 2.3. Antibacterial Properties against P. aeruginosa and MRSA

#### 2.3.1. Zone of Inhibition

**Figure 4:**
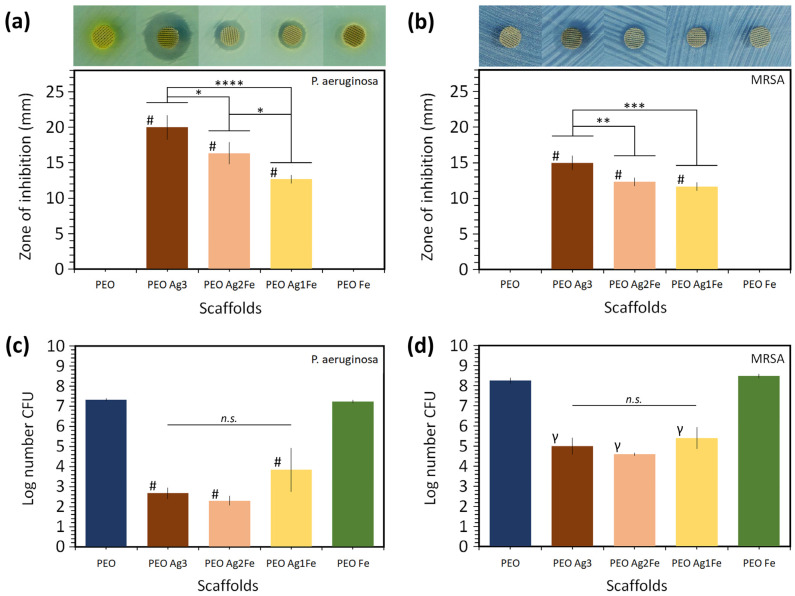
The zones of inhibition around the biofunctionalized scaffolds after 24 h of incubation on agar plates swabbed with a 10^7^ CFU/mL of (**a**) *P. aeruginosa* and (**b**) MRSA. The quantitative bactericidal activity of the scaffolds against (**c**) 2.6 × 10^6^ CFU of *P. aeruginosa* after 1 h of incubation and (**d**) 1.3 × 10^9^ CFU of MRSA after 4 h of incubation. **** = *p* < 0.0001, *** = *p* < 0.001, ** = *p* < 0.01, * = *p* < 0.05, # = **** vs. PEO and PEO Fe groups, γ = ** vs. PEO group and **** vs. PEO Fe group, n.s. = not significant.

Following 24 h of incubation, all the scaffolds containing Ag NPs (i.e., PEO Ag3, PEO Ag2Fe, and PEO Ag1Fe) showed inhibition activity against *P. aeruginosa* (Figure 4a) and MRSA (Figure 4b). On the contrary, such inhibition zones were not observed for the control group (i.e., PEO) and for the scaffolds containing Fe NPs only (i.e., PEO Fe). The PEO Ag3 specimens exhibited the largest sizes of the inhibition zones. The size of the inhibition zone was smaller for the specimens with lower Ag NPs concentrations (i.e., PEO Ag2Fe and PEO Ag1Fe).

#### 2.3.2. Bactericidal Activity

All the scaffolds bearing Ag NPs exhibited bactericidal activity against *P. aeruginosa* (Figure 4c) and MRSA (Figure 4d). The PEO Ag3, PEO Ag2Fe, and PEO Ag1Fe specimens demonstrated on average a 4-log, 4-log, and 3-log CFU reductions of *P. aeruginosa* after 1 h incubation (*p* < 0.0001 for all groups) as compared to PEO and PEO Fe specimens, respectively (Figure 4c). In addition, PEO Ag3, PEO Ag2Fe, and PEO Ag1Fe specimens exhibited on average a 4-log, 5-log, and 4-log CFU reductions of MRSA after 4 h of incubation as compared to PEO (*p* < 0.01) and PEO Fe (*p* < 0.0001) groups, respectively. The bactericidal activities of PEO Ag3, PEO Ag2Fe, and PEO Ag1Fe specimens against *P. aeruginosa* or MRSA showed no statistically significant differences.

### 2.4. Cytocompatibility

#### 2.4.1. Cell Proliferation and Morphology

**Figure 5:**
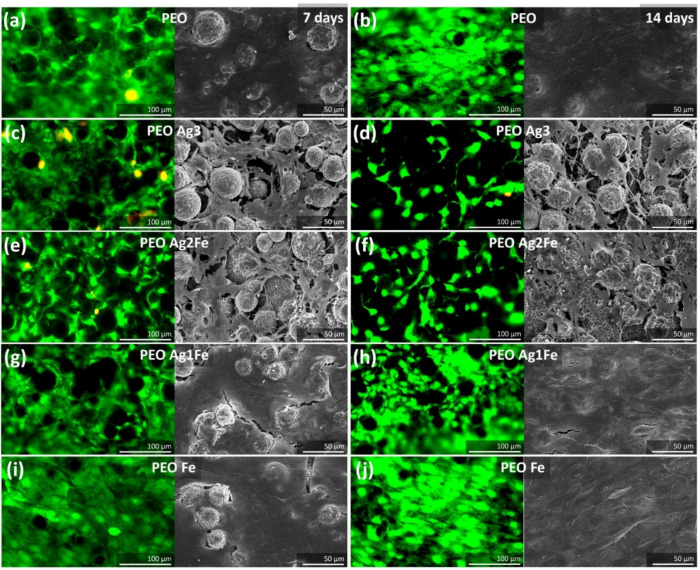
The cytocompatibility of the surface-biofunctionalized scaffolds towards preosteoblasts MC3T3-E1 under osteogenic conditions. The viability of cells was assessed by live/dead staining and the morphology of cells were imaged using SEM after culture for 7 and 14 days on the (**a**,**b**) PEO, (**c**,**d**) PEO Ag3, (**e**,**f**) PEO Ag2Fe, (**g**,**h**) PEO Ag1Fe, and (**i**,**j**) PEO Fe specimens.

The preosteoblasts showed favorable cellular behaviors, including adhesion and proliferation on PEO specimens (Figure 5a). After 14 days of culture, cells covered almost the whole surface of PEO scaffolds (Figure 5b). The inclusion of Ag NPs onto the surface layer influenced the proliferation of cells. Live/dead and SEM imaging revealed that after 7 days of culture, the cells in PEO Ag3 (Figure 5c) and PEO Ag2Fe (Figure 5e) groups could not yet cover the entire surface of the implants. On the other hand, after 7 days of culture, the cells on PEO, PEO Ag1Fe, and PEO Fe (Figure 5a, Figure 5g, and Figure 5i, respectively) groups formed at least a monolayer. Even after 14 days of culture, fewer cells were visible on PEO Ag3 and PEO Ag2Fe specimens (Figure 5d and Figure 5f, respectively) compared to other groups. The morphology of these cells was more spindle-like (Figure 5d and Figure 5f, respectively), whereas the cells on PEO Ag1Fe and PEO Fe specimens formed thicker cell layers fully covering the entire surface of the scaffolds (Figure 5h and Figure 5i, respectively).

#### 2.4.2. Immunostaining of Phospho-Calcium Sensing Receptors

**Figure 6:**
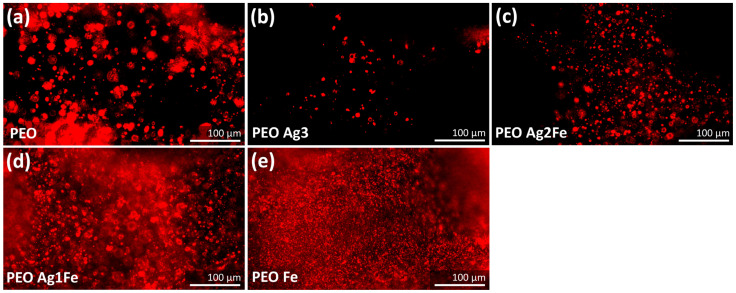
The immunostaining of phospho-CaSR in the preosteoblasts MC3T3-E1 after 14 days of culture on (**a**) PEO, (**b**) PEO Ag3, (**c**) PEO Ag2Fe, (**d**) PEO Ag1Fe, and (**e**) PEO Fe specimens.

After 14 days of culture under the osteogenic condition, the murine MC3T3-E1 cells phospho-CaSR were activated (Figure 6). Biofunctionalized surfaces with a higher concentration of Ag NPs showed less phospho-CaSR activation. The intensity of activated phospho-CaSR was the lowest on PEO Ag3 specimens (Figure 6b), followed by PEO Ag2Fe specimens (Figure 6c). A higher level of phospho-CaSR activation was observed on PEO Ag1Fe (Figure 6d) and PEO Fe (Figure 6e) specimens as compared to PEO specimens (Figure 6a).

## 3. Discussion

With the ever-increasing demand for (patient-specific) porous bone implants, made possible by AM, it is important to biofunctionalize the surface of implants to add antibacterial and osteogenic properties. We biofunctionalized the surface of SLM porous titanium using the one-step PEO process to create: (i) a surface morphology with a micro-/nano- porous feature that is known to favor bone cell adhesion and proliferation; (ii) surface chemistry composed of anatase and rutile phases that are enriched with bioactive Ca/P elements to promote bone regeneration; and (iii) a surface layer decorated with immobilized Ag and Fe NPs that continues to release Ag and Fe ions for up to 28 days. These morphological and chemical characteristics are essential for the whole biofunctionality package of a porous bone implant.

We explored herein the synergistic antibacterial properties of the titanium surface by immobilizing different concentrations of Ag NPs with a fixed concentration of Fe NPs. Surfaces decorated with Fe NPs and lower Ag NPs concentrations (i.e., PEO Ag2Fe and PEO Ag1Fe groups) exhibited comparable bactericidal activities against MRSA and *P. aeruginosa* compared to those with a higher Ag NPs concentration (i.e., PEO Ag3 group). In addition, the titanium surfaces bearing Fe NPs and the lowest Ag NPs concentration (i.e., PEO Ag1Fe group) allowed more preosteoblasts proliferation and better phospho-CaSR activation. Our findings confirmed the synergistic potential of Ag and Fe NPs on porous titanium surface—a strategy that is highly encouraging and warrants further development.

The failure of orthopedic implants is often caused by septic [38,39] or aseptic loosening [40]. The prevalence of implant-associated infection has been rising, as many pathogens have developed resistance against most of the available antibiotics [41]. Once infection sets in, osseointegration is hampered. Creating a multifunctional surface on bone implants is considered a promising strategy to fight multidrug-resistant microbial infections [42]. However, the development of such surfaces remains challenging. Such biofunctionalized surfaces are desired to both provide immediate as well as long-term protection against infection and simultaneously promote bone ingrowth for osseointegration [43]. Incorporation of antibiotics in the surface layer has been proven to be highly effective in the short term; however, the controlled release of antibiotics from antibiotic-engineered surfaces to treat infections in the long term is highly challenging and may even cause further development of antibiotic-resistant bacteria. In addition, biofunctionalized surfaces must adhere strongly to the substrate to survive the (forceful) bone grafting procedure. Antibiotic-free osseoinductive surfaces are promising candidates for addressing the issues of multidrug-resistant microbials and osseointegration [44,45]. Surface biofunctionalization by growing a porous TiO_2_ layer on the titanium substrate during PEO and incorporating Ca/P compounds and antibacterial NPs onto the oxide layer fulfills the desired dual functionalities [14,35,46].

The biofunctionalized surface presented here opens the possibility to immobilize Ag and Fe NPs in the Ca/P-enriched TiO_2_ layer that acts as a long-lasting reservoir to deliver antibacterial agents and bioactive agents. That is because the embedded NPs are not immediately released as particulates but are gradually oxidized into ionic compounds (e.g., up to 28 days in this study). Furthermore, the choice of antibacterial agents is of great importance to avoid the development of multidrug-resistant bacteria. The use of Ag NPs is a promising strategy, as bacteria may not acquire resistance to Ag NPs as quickly as to antibiotics [47], although it is not impossible [48]. Therefore, having multiple inorganic NPs altogether (e.g., Ag and Fe NPs) is expected to make it more challenging for bacteria to develop resistance.

Although Ag NPs have been extensively studied as an effective agent against multidrug-resistant bacteria [17,18,49], several in vivo studies have highlighted the adverse effects of Ag NPs on bone regeneration [20,21,22]. For this reason, we reduced the concentration of Ag NPs in the PEO electrolyte by 2- to 3-folds (i.e., PEO Ag2Fe and PEO Ag1Fe groups), which resulted in an overall decreased Ag ion release from the biomaterials (Figure 3a,b). A lower Ag ion concentration usually corresponds to reduced antibacterial activity [11,22,50]. However, our biofunctionalized scaffolds bearing Ag and Fe NPs (i.e., PEO Ag2Fe and PEO Ag1Fe groups) exhibited bactericidal activities similar to that of the PEO Ag3 group (Figure 4c,d). *P. aeruginosa* population was reduced by ≥3 orders of magnitude in all Ag-based scaffolds and those of MRSA by ≥4 orders of magnitude, after only 1 and 4 h of incubation, respectively (Figure 4c,d). Bacterial eradication by ≥3-log CFU within 18 h of incubation is considered to be at the level of bactericidal activity relevant to clinical practice [51]. On the other hand, the results of the agar diffusion assay did not show any synergistic potential of the biofunctionalized scaffolds (Figure 4a,b). All specimens with a higher Ag NPs concentration showed a larger inhibition zone, which was expected due to the disparity of the diffusion coefficients of Ag and Fe ions in the agar media. The PEO Fe scaffold group showed no zone of inhibition, indicating highly limited diffusion of Fe ions and/or limited bactericidal activity of Fe NPs.

We observed that the addition of Fe NPs led to higher antibacterial properties through a synergistic enhancement of the bactericidal properties of immobilized Ag NPs. The Ag and Fe NPs were clustered on PEO Ag2Fe and PEO Ag1Fe specimens (Figure 2f,g). This resulted in an enhanced Fe ions release (Figure 3c,d), which was triggered by local galvanic coupling, as Fe has a lower nobility than Ag and TiO_2_. Similar to our results, synergistic antibacterial behavior of Ag-Fe bimetals against *S. aureus* [52] and various multidrug-resistant Gram-positive and Gram-negative bacteria [53] has been reported.

Ag is not required in bacterial and cellular metabolism. The interaction of Ag with both mammalian and bacterial cells may cause damage to the cell membrane, cytoplasmic proteins, and DNA [54,55,56,57]. Therefore, Ag can be used as a very potent antibacterial agent. On the other hand, Fe is much less toxic to bacterial cells than Ag, which was proven by the results of the PEO Fe scaffold group (Figure 4 and Figure 5). Fe NPs require at least 2- and 8-fold higher concentrations than Ag NPs to inhibit multidrug-resistant *S. aureus* and *P. aeruginosa*, respectively [53]. That is because Fe is an essential nutrient for the metabolic activity and growth of most bacteria [58,59,60]. Nonetheless, Fe plays an important role in the catalysis of oxidative stress [61,62,63], which is one of the mechanisms utilized in the design of antibiotics to fight infections [64,65]. In addition to the synergistic metallic ion release, the generation of more reactive oxygen species when combining Ag and Fe NPs has also been reported [66]. Its contribution to combating multidrug-resistant bacterial infections is yet to be further explored.

In addition to exhibiting bactericidal behavior, biofunctionalized surfaces must allow host cells’ adhesion and proliferation. As expected, the surfaces bearing higher concentrations of Ag NPs (i.e., PEO Ag3 and PEO Ag2Fe groups) inhibited cell proliferation. At the lowest Ag NPs concentration (i.e., PEO Ag1Fe group), the proliferation of preosteoblasts was improved (Figure 5). To inhibit the growth of preosteoblasts, Fe ions require two orders of magnitude higher ion concentration as compared to Ag ions [67], which indicates that Fe is far less toxic to cells than Ag. This can be observed from the cells on the surface with embedded Fe NPs only (i.e., PEO Fe group), demonstrating no negative influence on cell adhesion and proliferation. During the PEO process, the corrosion of Fe NPs in the electrolyte might have occurred to some extent, releasing Fe ions that could form compounds with phosphate and become deposited in the porous oxide layer. Such phosphate-rich surface (Figure 2g) is known to favor osteoblast adhesion [32] and can be observed in the PEO Ag1Fe scaffold group, thus overcoming the toxicity of Ag NPs and improving the overall cytocompatibility of the scaffolds.

The phospho-CaSR activity indicates an early stage of osteogenic differentiation [68,69,70,71]. Active phospho-CaSR has been reported to positively influence alkaline phosphatase activity, osteocalcin expression, and mineralization [71]. The phospho-CaSR in the preosteoblasts was less active on the surfaces embedded with high Ag NPs concentrations (i.e., PEO Ag3 and PEO Ag2Fe groups). Usually, the phospho-CaSR in osteoblasts is stimulated when there is an elevated Ca ion concentration in the vicinity [72,73,74]. Among the biofunctionalized scaffolds, PEO Ag2Fe and PEO Ag1Fe groups released more Ca ions as compared to the control group (i.e., PEO, Figure 3). However, the lower activity of phospho-CaSR in the cells cultured on the PEO Ag2Fe group (Figure 6c) may be associated with relatively fewer cells present on these scaffolds (Figure 5f), as it is also the case for the PEO Ag3 group (Figure 5d and Figure 6b). Nevertheless, the underlying mechanism is not yet fully understood and needs further investigation. Taken together, the preosteoblasts cultured on PEO Fe and PEO Ag1Fe surfaces exhibited favorable osteogenic potential.

## 4. Materials and Methods

### 4.1. Scaffold Design and Selective Laser Melting

We designed porous titanium specimens (⌀ = 9.5 mm and h = 2 mm) with a strut size of 500 µm and a pore size of 350 µm (Figure 1a). Ti6Al4V ELI powder particles (medical grade 23, from AP&C, Boisbriand, QC, Canada) had a spherical morphology and particle sizes ranged between 10 and 45 µm. The specimens were selective laser melted (SLM-125, Realizer, Germany) using a YLM-400-AC Ytterbium fiber laser (IPG Photonics, Oxford, USA) under an Ar environment containing <0.2% O_2_. The SLM process parameters were as follows: laser current of 1100 mA, point distance = 10 µm, layer height = 50 µm, exposure time = 20 µs for the inner and outer boundary, exposure time = 5 µs for the hatch and the scanning strategy was 90° alternating. After SLM, loosely attached powder particles were removed from the scaffolds by vacuum cleaning, followed by ultrasonic cleaning in acetone, isopropyl alcohol, and demineralized water for 5 min each.

### 4.2. Plasma Electrolytic Oxidation (PEO)

The surface of the titanium scaffolds was biofunctionalized with the PEO in a calcium acetate (Dr. Paul Lohmann GmbH & Co, Germany) and calcium glycerophosphate (Sigma Aldrich, St. Louis, MO, USA) electrolyte. Ag NPs (colloidal, 65–75% basis, size: 7 to 25 nm, product no. 85131, Sigma Aldrich, St. Louis, MO, USA) and Fe NPs (nanopowder, 99.5% trace metal basis, size: 35 to 45 nm, product no. 746843, Sigma Aldrich, St. Louis, MO, USA) were dispersed in the electrolyte through ultrasonication for 5 min. Then, the electrolyte was stirred at 500 rpm for 5 min and ultrasonication was repeated for 5 min. The specimen groups and their corresponding electrolyte compositions with and without NPs are listed in Table 1. The PEO process was conducted using a custom-made laboratory setup (Figure 1d), including a double-walled glass electrolytic cell with stainless steel as the cathode and the scaffold as the anode, a thermostatic bath, an AC power supply (50 Hz, type ACS 1500, ET powder Systems, UK), and a computer interface connected to the power supply through a data acquisition board (SCXI, National Instruments, Austin, TA, USA). During the PEO process, the changes in voltage over time (*V-t*) were recorded at a sampling rate of 1 Hz. The surface biofunctionalization occurred under a galvanostatic condition using a current of 20 A/dm^2^ in the 800 mL electrolyte for 3 min, where the temperature was kept at <8 °C at the beginning and <20 °C at the end of the PEO process [11]. Thereafter, the surface-biofunctionalized scaffolds were rinsed with demineralized water and isopropyl alcohol for 5 min each at room temperature, sterilized at 120 °C for 2 h in an oven (Nabertherm TR60, NC, USA), and stored in a sterile environment.

### 4.3. Characterization of Surface Morphology, Porosity, and Phase Composition

The surface morphology of the biofunctionalized scaffolds was observed using a scanning electron microscope (SEM, JEOL JSM-IT100, Japan). The chemical compositions of the biofunctionalized surfaces were analyzed using an X-ray energy dispersive spectroscope (EDS) (JEOL JSM-IT100, Japan). The phase composition of the scaffolds was determined using an X-ray diffractometer (XRD, D8 Advance, Bruker, USA) with Bragg–Brentano geometry and a Lynxeye position-sensitive detector. The XRD analysis was conducted using Cu Kα radiation, at 45 kV and 40 mA, at a step size of 0.030°, and with a counting time of 2 s per step. The obtained XRD patterns were analyzed with the Diffrac Suite.EVA v5.2 software (Bruker, Billerica, MA, USA). The absolute porosity of the scaffolds was determined using Equation (1):

(1) $$\varphi =\left(1-\frac{m/\rho }{V_{bulk}}\right)\times 100\%$$

where *φ* is the absolute porosity [%], *m* is the mass [g] of the scaffold, *ρ* is the theoretical density of Ti6Al4V alloy (i.e., 0.00441 g/mm^3^), and *V_bulk_* is the bulk volume [mm^3^], calculated from the diameter and height of the scaffold specimen.

### 4.4. Release of Ag, Fe, and Ca Ions

The surface-biofunctionalized titanium scaffolds (in triplicate for each group for each time point) were immersed in 1 mL of phosphate-buffered saline (PBS) solution in a 48-well plate for 1, 4, 7, 14, and 28 days. The ion release experiments were performed in a static environment, at a temperature of 37 ± 0.5 °C, 95% relative humidity (RH), and in a 5% CO_2_ atmosphere. The PBS solution was collected and refreshed at each of the pre-selected time points. Ag, Fe, and Ca ion concentrations were determined by using inductively coupled plasma-optical emission spectroscopy (ICP-OES) (PerkinElmer Optima 3000DV, Belgium).

### 4.5. Antibacterial Assays

The antibacterial properties of the biofunctionalized scaffolds against *P. aeruginosa* (ATCC 27853) and MRSA (ATCC 33591) were investigated using agar diffusion plates and the counting of colony-forming units (CFU).

#### 4.5.1. Agar Diffusion

A 10^7^ CFU/mL bacteria suspension was prepared, following the McFarland standard [75]. The bacteria suspension was swabbed onto a minimal medium M9 agar plate for growing *P. aeruginosa* and a modified M9 agar plate for growing MRSA [76]. The biofunctionalized specimens (in triplicate for each group) were placed and pressed onto the agar plates to ensure sufficient surface contact for the diffusion of Ag and Fe ions. After 24 h of incubation at 37 °C, the agar plates were imaged, and the diameter of the inhibition zone was measured.

#### 4.5.2. Quantitative Bactericidal Activity

The surface-biofunctionalized scaffolds (in triplicate for each group for each time point) were filled with a 100 µL bacterial suspension (in physiological water) containing 2.6 × 10^6^ CFU of *P. aeruginosa* or 1.3 × 10^9^ CFU of MRSA in a 15 mL tube for 1 and 4 h, respectively. The CFU number of P. aeruginosa and MRSA was then determined by adding 400 µL of physiological water on the scaffold, mixed vigorously by vertexing, and spotted 5 µL of serial dilutions on Luria–Bertani agar plates, incubated for 24 h at 37 °C before colony counting.

### 4.6. Cytocompatibility

The cytocompatibility of the surface-biofunctionalized scaffolds towards preosteoblasts MC3T3-E1 was examined using live/dead staining assay and SEM imaging. In addition, immunofluorescence staining of phospho-CaSR Thr888 was performed.

#### 4.6.1. Preculture of Cells and Cell Seeding

Murine MC3T3-E1 preosteoblasts (Sigma Aldrich, Germany) were cultured in α-minimum essential medium (α-MEM, Thermo Fisher Scientific, Waltham, MA, USA) supplemented with 10% fetal bovine serum (FBS, Thermo Fisher Scientific, Waltham, MA, USA) and 1% penicillin/streptomycin (p/s, Thermo Fisher Scientific, Waltham, MA, USA) in a cell culture incubator at 37 °C, 95% RH, and 5% CO_2_. After reaching confluency, the cells were collected for experiments.

The preosteoblasts (5 × 10^4^ cells/specimen) were seeded and cultured on the biofunctionalized scaffolds in 6-well plates for 7, 14, and 21 days. An osteogenic cell culture medium made of α-MEM and supplemented with 10% FBS, 50 μg/mL ascorbic acid, 4 mM β-glycerophosphate, and 1% p/s was used from day two of cell culture onwards. The medium was refreshed every 2–3 days.

#### 4.6.2. Live/Dead Staining and SEM Imaging

After 7 and 14 days of cell culture, the specimens (in triplicate for each group for each time point) were placed in new wells and the viability of the cells was identified using calcein (green = live) and ethidium homodimer-1 (red = dead) staining (Thermo Fisher Scientific, Waltham, MA, USA). Thereafter, the viability and morphology of the cells were inspected using a fluorescence microscope (ZOE Cell Imager, Bio-Rad, CA, USA). In addition, the morphology of the cells on the specimens was observed using SEM (JEOL JSM-IT100, Japan). After 7 and 14 days of cell culture, the specimens were fixed using 4% formaldehyde (Sigma Aldrich, Germany), followed by dehydration steps in 70% and 100% ethanol and hexamethyldisilazane (Sigma Aldrich, Germany). The specimens were dried prior to imaging.

#### 4.6.3. Immunostaining

Phospho-CaSR Thr888 types of staining were performed on the surface-biofunctionalized scaffolds cultured with preosteoblasts at day 14 (in triplicate for each group). The specimens were fixed with 4% formaldehyde (Sigma Aldrich, Germany) and were permeabilized using 0.5% Triton/PBS (Sigma Aldrich, Germany). Then, the specimens were incubated with primary antibody rabbit polyclonal phospho-CaSR (1:100 per specimen, Thermo Fisher Scientific, Waltham, MA, USA) in 1% BSA/PBS, followed by washing with 0.5% Tween/PBS (Sigma Aldrich, Germany). The secondary incubation step was performed using rabbit Alexa Fluor 594 conjugated antibody (1:100, Thermo Fischer Scientific, Waltham, MA, USA) in 1% BSA/PBS, followed by washing with 0.5% Tween/PBS (Sigma Aldrich, Germany). Thereafter, the specimens were washed with PBS and were imaged using a fluorescence microscope (ZOE Cell Imager, Bio-Rad, CA, USA).

### 4.7. Statistical Analysis

The statistical analysis of the ion release results was performed with two-way ANOVA and a Tukey post hoc test. In addition, the statistical analysis of the antibacterial assay results was performed with one-way ANOVA, followed by a Tukey post hoc test (**** = *p* < 0.0001, *** = *p* < 0.001, ** = *p* < 0.01, and * = *p* < 0.05, n.s. = not significant).

## 5. Conclusions

Preventing implant-associated infections without delaying osseointegration requires the right type and doses of antibacterial agents. The biofunctionalized scaffolds developed in this study reduced the required Ag NPs concentration and demonstrated the synergistic potential of Fe and Ag NPs. The scaffolds biofunctionalized with three times fewer Ag NPs in the PEO electrolyte and exhibited comparable bactericidal activity against MRSA and *P. aeruginosa* compared to those of the scaffolds bearing three times higher Ag NPs concentration. Scaffolds with fewer Ag NPs but incorporating Fe NPs allowed preosteoblasts proliferation and phospho-CaSR activation in the cells at levels comparable to control groups (i.e., PEO without any NPs and PEO with Fe NPs only). Altogether, the biofunctionalization of porous titanium with Ag and Fe NPs is a promising strategy to prevent implant-associated infections, while simultaneously allowing osseointegration and bone regeneration. Such a strategy should be considered further for clinical applications.

## Figures and Tables

**Table 1:** Experimental groups and their corresponding compositions of the PEO electrolyte.

Specimen Group	Calcium Acetate (M)	Calcium Glycerophosphate (M)	Ag NPs (g/L)	Fe NPs (g/L)
PEO	0.15	0.2	-	-
PEO Ag3	0.15	0.2	3	-
PEO Ag2Fe	0.15	0.2	1.5	0.5
PEO Ag1Fe	0.15	0.2	1	0.5
PEO Fe	0.15	0.2	-	0.5

## Data Availability

Not applicable.
